# Annotations

**Published:** 1890-06-28

**Authors:** 


### Annotations.

"Heaven does with us as we with torches do; not light them for themselves." We may presume also that the wealth, beauty, and unlimited variety of our English flowers were not fashioned and coloured solely for their own delight. It cannot perhaps be said with truth of the wealthy persons who live in rural districts, as it was of those in the parable who despised the feast to which they were bidden, that they do not enjoy very profoundly the delight of living among the flowers by which they are daily surrounded. But it is true that they cannot feel the same rapturous pleasure at the sight of the familiar faces of the lawn and the hedgerow as do the thousands of poor little town children who are suddenly transported from their hard pavements, their miserable houses, and their skyless streets to the woods, the meadows, and the delightful gardens of the country. Just now, thanks to the recent rains and warmth, the roses are bursting into full bloom, the rhododendrons are at the height of their perfection, the foxgloves are brilliant, the honeysuckle is scenting the morning and evening air, and the ten thousand little denizens of the meadows and the banks are adding their quota of perfume and of colour. They all seem to unite in the very words of the parable and to say, "Go ye out into the slums and alleys of the cities and compel all the poor, pale-faced children to come and behold us; for though thousands of us have already faded and died, there are thousands yet in full bloom, and thousands more are ready to burst forth." The various societies, clergy, and missionaries are sending their summer appeals to the newspapers for means to give the children for whom they specially concern themselves "a day in the country." Those whose business takes them daily to London see morning by morning long excursion trains filled with happy children, who crowd the carriage windows, shouting and waving hats and handkerchiefs, and otherwise doing their best to show to the world the exuberant delight of their hearts. A day in the country, it may be admitted, is not so valuable for health as two or three weeks in a village home. But when the choice lies between a single day's excursion and no peep of the country at all during the long, hot days of summer, there can be no hesitation in deciding that "half a loaf is better than no bread." We ask our readers to scan carefully in their morning papers the column which is headed "A Day in the Country," and having chosen that particular school, or society, or mission which they think most necessitous or most deserving, to give to it with generous liberality, in order that no boy or girl may be left behind when all the school starts on its happy excursion. The two or three shillings which so many can easily spare, may make the brightest day of a lifetime to an East-end child.

**The Flowers are Calling.**

Under the heading of "Ethnological Notes," a medical contemporary reminds us of certain facts brought to light some years ago by Mr. Joseph Thompson concerning a peculiar race of Africans. "The Masai tribes," says our contemporary, "who inhabit the rich volcanic country east of the Victoria Nyanza, are among the most interesting people in Central Africa. They are not negroes: they have a good cranial development, straight, well-shaped noses, eyes with a slight Mongolian slant upwards, prominent cheek-bones, jaws rarely prognathous, chocolate-coloured skins, and extremely well-proportioned limbs. . . . The chief achievements of the young Masai men or warriors seem to be to make eloquent speeches and to commit murder. Internecine wars have depopulated their country, and their ferocious attacks on caravans have made Masai Land both the terror and the *terra incognita* of travellers. . . . The married men and women live in kraals apart, and the unmarried girls and warriors live together in other villages by themselves in a state of promiscuous free love. The women are completely clothed in dressed hides, and are as immoral as the men are arrogant and ferocious. . . . They have no religion, and no belief in a future." These facts may be commended to the consideration of Mr. Grant Allen and any persons who may be in sympathy with his lately-expressed views on the racial advantages of free love and the immediate gratification of mutual passion. It is evident that Mr. Allen has been anticipated in his advanced philosophy by the Masai. All scientists prefer induction to deduction, and a concrete fact to a plausible theory. In Masai land the youthful Hercules with a big brain and nobly proportioned limbs may, without let or hindrance from marriage laws or conventional tastes, take his Venus or his Cleopatra whenever he can persuade her to become his own. The result of this is before us in the peculiar endowments of the Masai tribe. Eloquence of tongue is one of them, and a keen relish for indiscriminate murder is another. Their hospitality to strangers is shown by their extreme readiness to deliver their visitors from the sorrows and troubles of life by giving them the happy dispatch. It is not stated that there are any poets among them, or that they have built up a literature. No mention is made of their scientific achievements, or of their high distinction in art. Philosophy and medicine seem to be unknown in their country. Looked at comparatively they appear to be somewhat lower in the scale of development than the domestic dog or the uninstructed chimpanzee. We make Mr. Grant Allen a present of them. If he believes in his own theories, he will probably take up his residence in Masai land; in which case he will at least be sure of advanced and congenial society. For our part, judging from the experience of the last two centuries, we are inclined to think that only one thing is wanted to turn these murder-loving and bestial creatures into wholesome, intelligent, and kindly men and women; and that one thing is the Christian religion. Even Mr. Grant Allen would not deny that if those terrible people could be persuaded to accept the pure religion of Christ, they would be changed at once from savage and lustful beasts into reasonable creatures.

**Wanted, a Religion.**

Professor Bonney was one of the speakers at the annual gathering of the past and present students of the Queen's College for Ladies, Harley Street, held last Saturday. The Professor declared, among other things, that over-specialisation was becoming a dangerous thing in science. Every day's experience proves this true. What is specialisation? It is the concentration of the mind, or it may be of the hand, upon one strictly limited subject, with the view of mastering that subject with perfect thoroughness. That concentration is manifestly a desirable and a necessary thing. With such specialisation no scientific man, and no really intelligent man in any department of activity, can find the least fault. But what, then, is over-specialisation? It is not merely the concentration of the mind on one particular subject, but it is the devotion of the mind to that subject exclusively. And wherein lies the danger of over-specialisation as thus defined? It lies in two or three directions. In the first place the scientist himself is injured; and in the next his department of science is either dwarfed or magnified unduly. It is seen out of proportion by the scientist himself; and it is presented by him to the world in other than its true relative dimensions and importance. Thus, in the third place, all the world except the very few whose general knowledge is extensive enough, and whose intellect is adequate to the rational measuring of the various phenomena that engage the attention of man, are misled and often grievously bewildered without reason and to their serious mental hurt. But it is the specialist himself who is most disadvantaged. Giving up his intellect exclusively to one subject, and that often extremely limited in its own nature, he soon masters practically all that can be known about it; and then he begins to deal in what may be called "the curiosities" of his specialty. The subject is entirely exhausted so far as the necessity for strenuous mental labour is concerned, and so its exclusive devotee becomes lazy and, intellectually, quite inconsequent. Having neglected all other departments of study, he now either fears or despises them. He looks upom them as competitors with his own hobby, and as competitors which, though far behind in real importance, may yet by some unlucky chance be pushed to the front and leave him and his particular specialty out of sight. The mere specialist, whether he be biologist, chemist, theologian, or pointer of steel pens is of exceedingly small account. The only specialists who have ever been of weight and significance to the great world have been those who, to a thorough knowledge of their specialty, have added a general knowledge of man: of man in the present, and of man in the past: of man the thinker, man in his relation to God, man in his relation to his all-pervading environment. The specialist who is a specialist only is as impotent in a world of living and active men as is a toy boat when fleets are wanted for the carriage of the armies or the merchandise of a great country.

**Over-Specialisation.**

